# Development of a Compact NDIR CO_2_ Gas Sensor for a Portable Gas Analyzer

**DOI:** 10.3390/mi15101203

**Published:** 2024-09-28

**Authors:** Maosen Xu, Wei Tian, Yuzhe Lin, Yan Xu, Jifang Tao

**Affiliations:** 1 College of Electrical Engineering and Automation, Shandong University of Science and Technology, Qingdao 266590, China; xumaosenld@163.com; 2 College of Electronic and Information Engineering, Shandong University of Science and Technology, Qingdao 266590, China; 3 School of Information Science and Engineering (ISE), Shandong University, Qingdao 266237, China; memst1an@163.com (W.T.); linyz@sdu.edu.cn (Y.L.)

**Keywords:** CO_2_ sensor, NDIR, MEMS emitter, air quality monitoring

## Abstract

A carbon dioxide (CO_2_) gas sensor based on non-dispersive infrared (NDIR) technology has been developed and is suitable for use in portable devices for high-precision CO_2_ detection. The NDIR gas sensor comprises a MEMS infrared emitter, a MEMS thermopile detector with an integrated optical filter, and a compact gas cell with high optical coupling efficiency. A dual-ellipsoid mirror optical system was designed, and based on optical simulation analysis, the structure of the dual-ellipsoid reflective gas chamber was designed and optimized, achieving a coupling efficiency of up to 54%. Optical and thermal simulations were conducted to design the sensor structure, considering thermal management and light analysis. By optimizing the gas cell structure and conditioning circuit, we effectively reduced the sensor’s baseline noise, enhancing the overall reliability and stability of the system. The sensor’s dimensions were 20 mm × 10 mm × 4 mm (L × W × H), only 15% of the size of traditional NDIR gas sensors with equivalent detection resolution. The developed sensor offers high sensitivity and low noise, with a sensitivity of 15 μV/ppm, a detection limit of 90 ppm, and a resolution of 30 ppm. The total power consumption of the whole sensor system is 6.5 mW, with a maximum power consumption of only 90 mW.

## 1. Introduction

Carbon dioxide (CO_2_) is a common colorless and odorless gas that plays an important role in both production and daily life. CO_2_ is a byproduct of the human metabolic cycle and reflects the physiological state of the body [1,2,3]. Therefore, respiratory gas detection is crucial for health diagnostics in clinical practice [4]. The concentration of exhaled CO_2_ is used to confirm whether a respiratory catheter is correctly placed in the trachea [5]. CO_2_ is also a key indicator of indoor air quality, with an atmospheric concentration of approximately 0.043%. When indoor CO_2_ concentration increases to 3%, people may experience an accelerated heartbeat and rapid breathing. At 5%, symptoms such as difficulty breathing and tinnitus can occur [6]. Therefore, monitoring and alerting for indoor air quality are essential for ensuring people’s health [7].

Compared to other gas sensors, NDIR gas sensors offer high reliability, fast response times, high selectivity, and low cost. With the advancement of MEMS technology, significant progress has been made in the miniaturization of NDIR CO_2_ sensors. In 2016, Vincent et al. [8] developed a handheld CO_2_ sensor for a portable breath analyzer. The sensor achieved an accuracy of 2.9% with a measurement range of 0.5% to 4%. In 2017, Seung et al. developed a CO_2_ gas sensor with a double-ellipse sampling gas chamber that increased the infrared light transmission path and improved optical coupling efficiency to 48%. However, the chamber structure was complex and large [9]. In 2020, Vafaei et al. proposed a cavity-free NDIR method to measure CO_2_ concentrations between 400 ppm and 2200 ppm [10]. In 2021, Jia et al. [11], from Ghent University in Belgium, developed a fully integrated NDIR carbon dioxide sensor implemented on a silicon chip. The light source, detector, and cylindrical gas chamber were all integrated on the chip, with the sensor occupying only 7 mm^2^ in volume. In 2022, Jing Yang et al. [12] proposed a highly integrated, compact infrared gas sensor design with dimensions of only 10 × 10 × 1 mm^3^. The gas cell was formed using two silicon wafers processed and bonded together through wafer bonding. However, the optical gas chamber had a low coupling efficiency of only 17.6%. In 2023, Zhang et al. [13] proposed and validated a novel planar conical cavity NDIR CO_2_ gas sensor capable of accurately detecting concentrations from 0 to 2000 ppm at 25 °C. Although NDIR CO_2_ gas sensors have made significant advancements, challenges persist, including the complexity of gas chamber structures, low coupling efficiency, and relatively low sensitivity, which need to be addressed.

To address issues such as low sensor sensitivity and resolution, we conducted research on the design of the gas chamber structure and the infrared optical coupling system based on optical and thermal simulation analyses of the sensor. By optimizing the circuit design, we successfully developed a high-sensitivity, low-noise miniature infrared CO_2_ gas sensor and completed its performance validation. The sensor’s dimensions were 20 mm × 10 mm × 4 mm (L × W × H), only 15% of the size of traditional NDIR gas sensors with equivalent detection resolution. The sensor sensitivity was 15 μV/ppm, the response time was 3 s, the detection limit was 90 ppm, and the detection resolution was 30 ppm.

## 2. Design and Simulation of the Sensor

**Figure 1:**
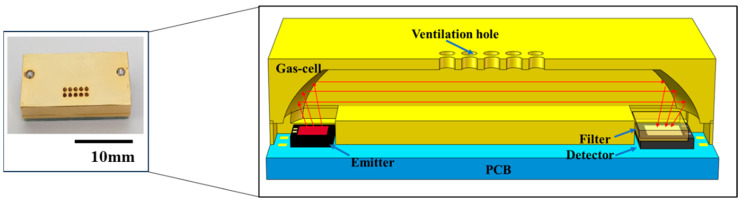
The structure of the sensor. The red arrow represents the direction of light propagation.

The design of a CO_2_ sensor with the potential to be integrated into a potable gas analyzer is presented here. The NDIR gas sensor was composed of key components, including a MEMS infrared emitter, a gas chamber, a thermopile detector, and a filter with a central wavelength of 4.26 µm (Full Width at Half Maximum (FWHM) = 0.09 µm). The main parameters of the thermopile detector in this manuscript were as follows: the resistance was 71 kΩ, the sensitivity was 45 V/W, the noise voltage was 47.2 nV/√Hz, and the response time was 15 ms. In our design, the MEMS emitter was a blackbody radiation emitter with a central sensitive area of 1.2 mm × 1.2 mm, maintaining a modulation depth of 100% within 20 Hz [14]. The overall dimensions were 1.8 mm × 1.8 mm × 0.4 mm, with a central radiation area of 1 mm × 1 mm. In this work, both the MEMS emitter and detector were bare chips within the sensor, as shown in Figure 1. Compared to traditional NDIR gas sensors, which integrate pre-packaged chips, this approach significantly reduces the sensor’s size. The overall dimensions of the sensor were 20 mm × 10 mm × 4 mm. For comparison, the size of a traditional NDIR sensor, such as the CUBIC NDIR CO_2_ gas sensor, is 33 mm × 19 mm × 9.4 mm [15], which represents the standard size for conventional NDIR gas sensors. The sensor developed in this work was only 15% of the traditional sensor’s size.

According to NDIR detection principles, the reduction in light intensity caused by gas absorption adheres to the Beer–Lambert Law [16]:

(1) $$I=\eta I_{0}e^{-\alpha CL}$$

where *η* represents the optical coupling efficiency, *I* and *I*_0_ denote the light intensities at the end and beginning of the optical path, respectively, *L* is the optical path length through the gas cell, *α* is a constant dependent on the specific wavelength of the infrared light used, as well as the temperature and pressure of the gas sample, and *C* is the concentration of CO_2_ in parts per million (ppm). For signals using a thermopile detector, the formula for calculating the change in output voltage is as follows:

(2) $$NA=\frac{\left(V_{0}-V\right)}{V_{0}}=\frac{\left(I_{0}-I\right)}{I_{0}}=1-\frac{I}{I_{0}}$$

where *NA* is the normalized absorption rate, *V*_0_ is the amplitude voltage of the incident light intensity *I*_0_ received by the detector, and *V* is the amplitude voltage of the light intensity *I* after passing through the gas, as received by the detector. According to Equations (1) and (2):

(3) $$NA=1-e^{-\alpha CL}$$

According to the formula, it can be observed that when the gas concentration is low, the absorption rate of the gas is higher, resulting in a larger signal change. By adjusting the optical path length *L* according to the range of CO_2_ concentration, infrared light can be better absorbed.

**Figure 2:**
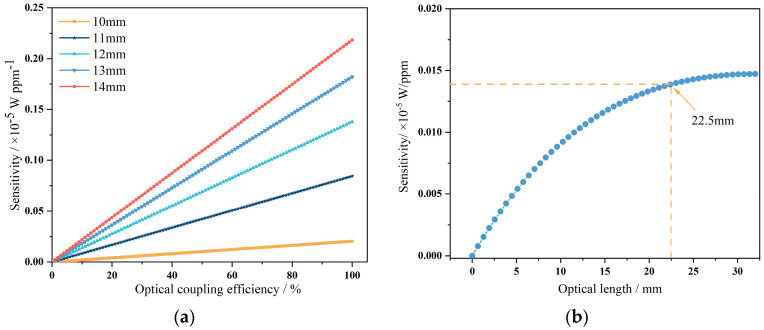
(**a**) The impact of coupling efficiency on sensitivity at different optical path lengths. (**b**) The variation in sensitivity with optical path length.

Based on Lambert–Beer’s Law [16], it is possible to derive the relationship between coupling efficiency, optical path length, and the sensitivity of the sensor [17]. The enhancement effect of coupling efficiency on sensitivity at a concentration of 400 ppm with a 10–14 mm optical path was analyzed. As shown in Figure 2a, it was observed that at shorter optical path lengths, the improvement in sensitivity due to coupling efficiency was relatively limited. However, as the optical path length increases, the impact of coupling efficiency on sensitivity becomes more pronounced. To enhance the sensor’s sensitivity, it is necessary to increase the optical path length while maintaining high optical coupling efficiency. From Figure 2b, it can be seen that at low concentrations, when the optical path length exceeds 22.5 mm, the sensitivity improvement with increasing optical path length becomes limited. Moreover, a longer optical path length leads to an increased number of reflections, resulting in reduced light intensity and making light detection more challenging. Therefore, to enhance sensitivity, it is essential to ensure that the optical path length is at least 22.5 mm in addition to improving coupling efficiency.

### 2.1. Gas Chamber Design and Assembly of Components

**Figure 3:**
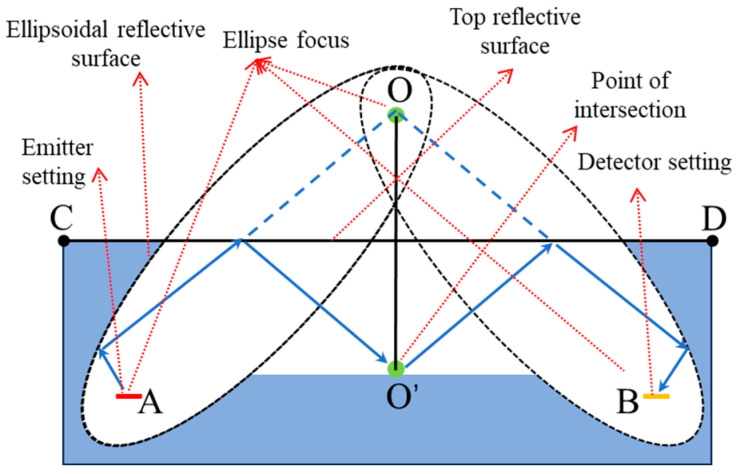
Optical path of the gas cell.

To improve the sensor’s sensitivity, it is necessary to achieve an optical path length of no less than 22.5 mm while maximizing coupling efficiency. Given the MEMS infrared light source’s divergence angle of ±45°, a specially designed structure must be used as a reflector to shape the light propagation and improve the detector’s optical coupling efficiency. An innovative dual-ellipsoid mirror optical system was designed and the specific light path is shown in Figure 3. The gas chamber was composed of three parts: the top surface, the bottom surface, and the ellipsoidal reflective surface. The light emitted from the light source illuminates the ellipsoidal surface, reflects onto the bottom surface of the gas chamber, and then converges at the detector through the ellipsoidal surface above the detector. The light absorbed by the detector in the gas chamber space mainly comes from the reflected light of the ellipsoidal surface, which has already been reflected 3–5 times, thereby increasing the optical path length. In this design, the emitter and detector of the sensor are located at the focal points A and B of the ellipses. O’ is the symmetric point of the ellipse’s focal point O. CD is the perpendicular bisector of OO’.

**Figure 4:**
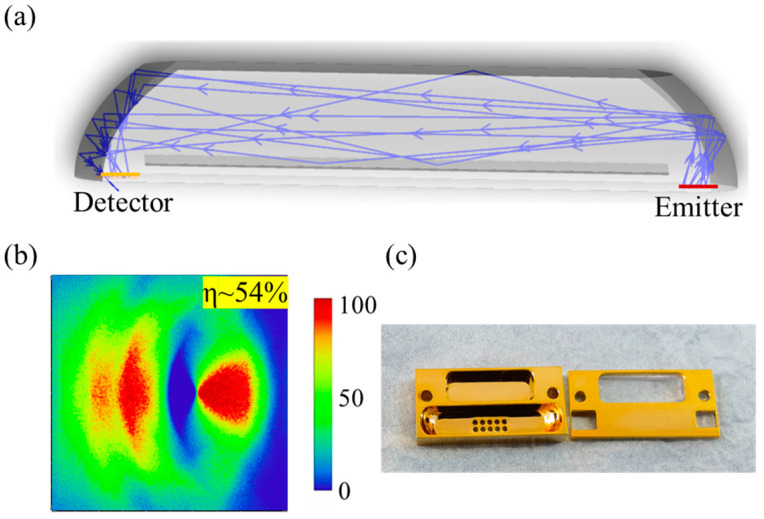
(**a**) Optical reflection path of the gas cell. (**b**) The light spots on the detector simulated by ray tracing. (**c**) The physical image of the gas cell with a thin gold layer of 200 nm.

An optical path simulation model was constructed using the ray tracing method, as depicted in Figure 4a. The parameter settings are shown in Table 1. According to the ray tracing simulation, the coupling efficiency of the gas chamber was 54%, as shown in Figure 4b, and the average optical path length was 24 mm.

The optical coupling efficiency of this design was substantially improved, being about 5–8 times greater than that of conventional NDIR gas sensors, which usually have optical coupling efficiencies between 5% and 10% [12], as illustrated in Figure 4c. To minimize light reflection losses during the reflection process, the surface of the gas cell was polished and coated with a 200 nm thin layer of gold [18]. This can reduce surface roughness and improve reflectivity in the mid-infrared range [19,20]. According to research, this combination of polishing and gold coating has achieved a reflectivity of over 95% [19]. In addition, gold also has good chemical stability, which can enhance the stability and reliability of the sensor. The dimensions of the gas cell were 20 × 10 × 3 mm^3^.

### 2.2. Thermal Simulation

**Figure 5:**
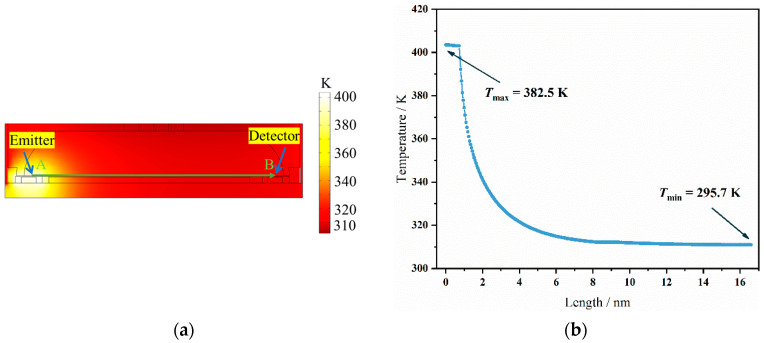
(**a**) The temperature distribution of the sensors using the FEM. (**b**) The temperature curve from the emitter to the detector.

In this work, the light source was a MEMS infrared emitter based on blackbody radiation, which operates at temperatures exceeding 400 °C. The MEMS thermopile detector, on the other hand, is highly sensitive to temperature changes. Therefore, managing the high temperature of the MEMS infrared emitter during operation presents a challenge for thermal management within the integrated sensor. To analyze whether the operation of the MEMS emitter affected the detector, a thermal simulation model of the sensor was established using the finite element method (FEM), analyzing the temperature distribution throughout the sensor when the MEMS infrared emitter was in operation. In the model setup, the initial temperature was set to 20 °C, and it was assumed that the boundary temperature at the top of the enclosure was also 20 °C. The results are shown in Figure 5a. Once the system stabilized, the temperature was significantly higher only at the MEMS emitter, whereas the temperature near the detector was close to room temperature. To study the temperature variation from the MEMS emitter to the detector in detail, probes were placed along the green dashed line from the MEMS emitter to the thermopile detector to analyze temperature changes. The temperature distribution curve from the emitter to the detector is shown in Figure 5b. The results indicate that when the system reaches steady state, the highest temperature (382.5 K) occurs at the MEMS emitter. The temperature gradually decreased as the distance from the light source increased, reaching the lowest temperature of 296 K at the detector, slightly above the ambient temperature of 293 K.

According to Jones’ theory [21], there are three different types of noise in thermal detectors: temperature noise, thermal noise (also called Johnson noise), and signal noise. The first two are the primary noise sources for thermopile detectors. However, when the system stabilizes at room temperature and the detector’s operating temperature is close to room temperature, thermal noise is at least five times greater than temperature noise caused by temperature fluctuations [22]. Therefore, at room temperature, the impact of temperature noise on the detector is negligible, and thermal noise is the predominant noise. Thermal noise can be calculated using the following equation [23]:

(4) $$V_{rms}=\sqrt{4kTR\Delta \upsilon }$$

where Δ*v* is the measurement frequency bandwidth, *k* is the Boltzmann constant, *T* is the absolute temperature, and *R* is the resistance of the thermopile detector, which is 71 kΩ. The thermal noise of the detector, as determined by simulation, was 0.01 μV.

### 2.3. Detector Signal Processing Circuit

**Figure 6:**
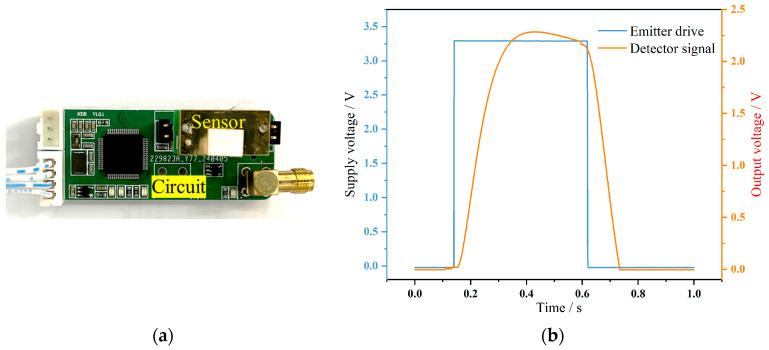
(**a**) The hardware circuit with a gas sensor. (**b**) The signal response curve of the detector with respect to the MEMS emitter.

Since the MEMS emitter is an active device, it requires a pulse voltage to operate at a certain frequency, causing the detector to produce a pulse signal with the same frequency as the emitter. According to the evaluation of the MEMS emitter, the operating voltage of the emitter is 3.3 V, and the pulse width must not be less than 15 ms [14]. Therefore, as shown in Figure 6a, a hardware circuit needs to be designed to drive the MEMS light source and process the small signal from the detector. The signal directly output by the detector is typically in the μV range, making it difficult for general data acquisition equipment to capture. In detection, due to noise signal masking, conventional demodulation and other detection schemes are not feasible as these methods cannot significantly improve the signal-to-noise ratio and cannot extract the effective signal from the noise. Through verification, it was found that narrow-band filtering and signal amplification can achieve signal extraction and processing. As shown in Figure 6b, based on the characteristics and requirements of the thermopile voltage output, a filtering and amplification circuit was designed to process the detector’s signal amplitude to measure its peak-to-peak value. By using a two-stage operational amplifier and narrow-band filtering, the signal gain was designed to be 5000, with a passband range of 0.2 to 5 Hz. The attenuation strength of the 50 Hz interference signal was tested to be −10 dB.

Based on testing, the total power consumption of the hardware circuit was only 4 mW, whereas the infrared light source operated at 400 °C with a power of 170 mW. To reduce power consumption, a pulse operation mode was used, with a pulse frequency of 1 Hz and a duty cycle of 50%. In this mode, the average power of the light source was 85 mW. In practice, the pulse width of the light source can be reduced to 15 ms, and the duty cycle can be decreased to 1.5%, resulting in an average power of 2.5 mW. By applying an even lower pulse frequency, further power reduction can be achieved. The total power consumption, including the sensor and circuit, was 6.5 mW, with a maximum power consumption of only 90 mW. This is significantly lower than traditional NDIR gas sensors (typically 100–150 mW), meeting the power requirements for portable analyzers.

## 3. Experimental Results and Discussions

**Figure 7:**
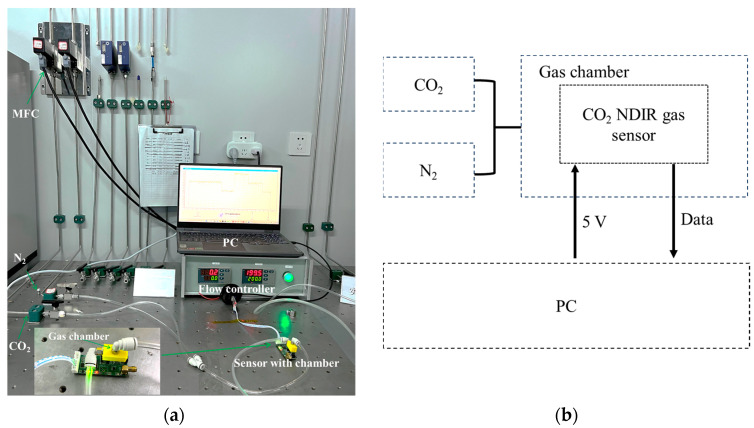
Gas sensor testing platform. (**a**) Photograph of the experimental setup. (**b**) Block diagram of the testing platform.

To evaluate the sensor’s ability to monitor CO_2_ concentration, the prototype of the miniature NDIR CO_2_ gas sensor was tested and assessed. This involved accurately controlling the gas concentration within a predetermined measurement range to precisely evaluate the measurement accuracy of the prototype at specific concentration points. The setup for testing the gas sensor is shown in Figure 7. The gas is controlled using two mass flow controllers (MFCs, model: Xinnovis MFC-T), each connected to two gas cylinders: one containing 99.99% pure N_2_ gas and the other containing 10% standard CO_2_ gas. The sensor testing apparatus included a sensor test gas chamber, the gas sensor, and the associated circuitry. Data collection and storage were managed via computer software on an upper-level machine.

**Figure 8:**
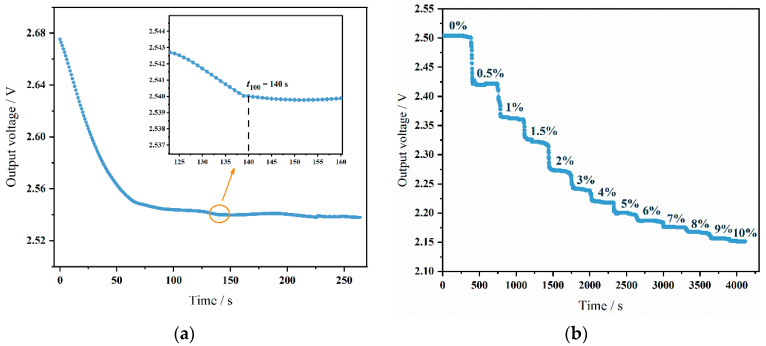
(**a**) Sensor system power-up time. (**b**) The dynamic response curve of the sensor.

Based on the constructed gas sensor testing system, the sensor’s performance was evaluated. Different volumes of N_2_ and CO_2_ were controlled using flow controllers to achieve CO_2_ concentrations ranging from 0% to 10%. Testing different CO_2_ concentrations assessed the sensor’s response. Across the whole experiment, the gas flow was maintained at a constant rate of 100 sccm. The initial power-on time of the sensor was tested first to evaluate the time required for the sensor to reach optimal operational status after startup. Testing in a pure N_2_ environment yielded results, as shown in Figure 8a. The sensor reached a stable output state 140 s after powering on, indicating a total response time (*t*_100_) of 140 s. Through these tests, the sensor’s response characteristics at different time points were clearly understood, which is crucial for optimizing startup time in practical applications and improving system response efficiency. Additionally, knowing the sensor’s response time under specific conditions helps in designing control systems and setting alarm thresholds to ensure safety and reliability under various operational conditions.

The sensor’s response to different concentrations was tested. CO_2_ gas concentrations were divided into 0%, 0.5%, 1%, 1.5%, 2%, 3%, 4%, 5%, 6%, 7%, 8%, 9%, and 10%, comprising 13 concentration points. Each concentration point was maintained for 5 min after stabilization. Sensor response was assessed by monitoring voltage changes at varying CO_2_ gas concentrations. The sensor’s dynamic response at different concentrations is depicted in Figure 8b. The tests indicate that the sensor effectively distinguishes between all 13 concentration points. Benefiting from the optimized chamber design and circuitry, the sensor shows a strong response at low concentrations. Optimizing the chamber design to extend the optical path can significantly enhance sensor sensitivity at lower concentrations. However, this may reduce resolution at higher concentrations.

**Figure 9:**
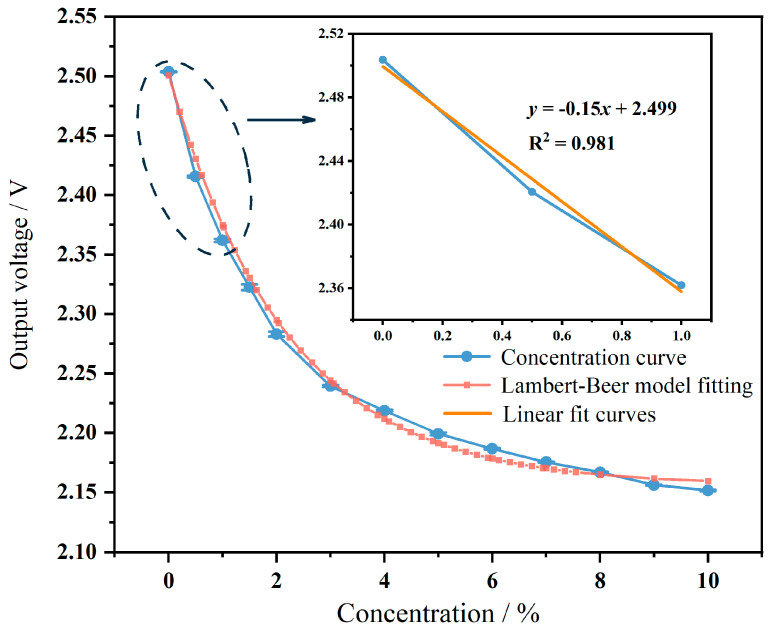
The sensitivity curve of the sensor.

The sensitivity of the sensor can be determined by the concentration value and the change in signal response. Multiple measurements of the sensor signal at each concentration point yield the concentration change curve of the sensor, as shown in Figure 9. The slope of the curve represents the sensitivity of the sensor at different concentrations. From the graph, it can be observed that as the concentration increases, the sensitivity of the sensor gradually decreases, which conforms to Lambert–Beer’s law. However, at low concentrations, the concentration change is linear, indicating approximate linearity of sensitivity. From the analysis of the inset in Figure 8, within the concentration range of 0 to 1%, the linear regression function is *y* = −0.15*x* + 2.499, with a goodness of fit (R^2^) of 0.981, indicating good linearity of concentration change from 0 to 1%. Therefore, its slope represents the sensor’s sensitivity, which is 15 μV/ppm in the sensitivity range of 0 to 1%. Compared to previous work [24], although the size slightly increased, the sensitivity improved nearly threefold.

**Figure 10:**
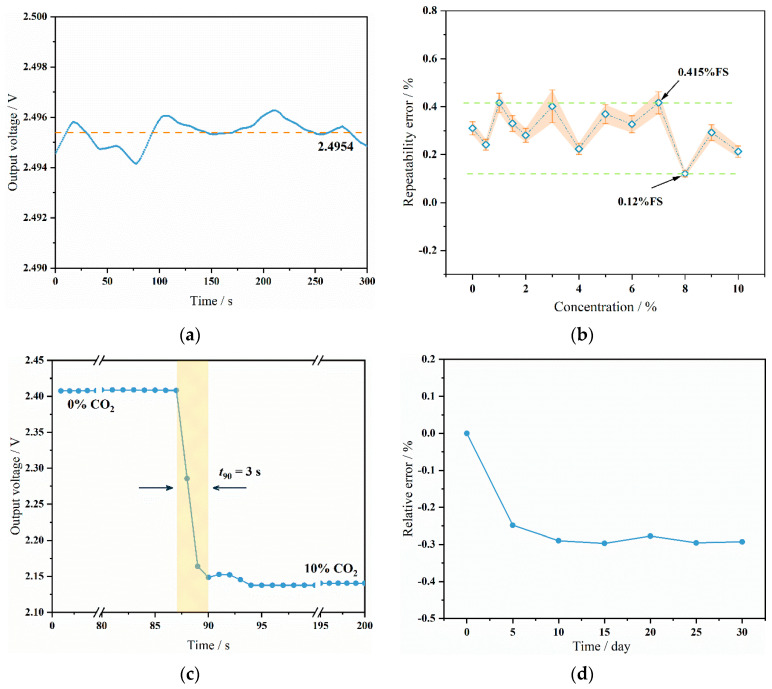
(**a**) The noise testing curve of the sensor (The dashed line represents the true value). (**b**) Repeatability errors at the same CO_2_ concentrations. The dashed line indicates the maximum and minimum values of the error, while the shaded area represents the range of data error from the tests. (**c**) The response curve of the sensor. (**d**) The stability curve of the sensor over the long-term.

The detection limit (LOD) is a critical parameter that should be considered when comparing sensor performance. Analyzing the detection limit requires calculating the system’s background noise. The formula for calculating the detection limit is as follows:

(5) $$\mathrm{LOD}=3\frac{{rms}_{noise}}{S}$$

where *rms_noise_* represents the background noise of the system and *rms* noise indicates the root mean square deviation of the signal baseline at zero concentration [25]. Typically, three times the effective amplitude of the measured noise (3*rms_noise_*) is used as the background noise of the sensor. *S* is the sensitivity of the sensor, which is 15 μV/ppm. To determine this, testing was conducted on the sensor’s background noise. The sensor operated under pure nitrogen conditions for a period of time, and the results are shown in Figure 10a. The sensor’s signal baseline was 2.4954 V, the root mean square deviation of the sensor’s signal baseline was calculated to be 0.471 mV, and three times the baseline noise signal was 1.44 mV. Using Formula (2), the sensor’s detection limit was calculated to be 94 ppm, with a resolution of 30 ppm.

In addition to the detection limit and sensitivity, the repeatability error of the sensor is vital for its measurement accuracy. The repeatability and stability of the sensor were assessed. The sensor underwent 10 tests at each concentration level to evaluate its repeatability performance. The repeatability error at each concentration is depicted in Figure 10b. The results indicate that the sensor’s repeatability error ranges from 0.12%FS to 0.415%FS (%FS stands for ‘percent of full scale’). This refers to the percentage of the sensor’s full measurement range used to express the error. This demonstrates that the sensor exhibits high repeatability and reliable detection performance. Such consistent performance across multiple tests confirms the sensor’s reliability and stability, making it suitable for precise CO_2_ concentration measurements in various applications. The response time is also a crucial performance indicator for sensors. Therefore, this test evaluates the sensor’s response time by switching the concentration between pure N_2_ and 10% CO_2_ standard gas, maintaining the same gas flow rate as before. The results are shown in Figure 10c, where the sensor’s response time *t*_90_ is 3 s, and *t*_100_ is 6 s.

The signal drift of the sensor over a long-term operation period was also evaluated. The sensor operated continuously under pure nitrogen conditions for 30 days, with signal data collected every five days to analyze its drift over prolonged operation. The test results are shown in Figure 10d. Initially, the sensor signals fluctuated significantly, but over time, the signal gradually stabilized and no longer exhibited substantial changes. After one month, the signal deviation from the initial signal was −0.27%, corresponding to a concentration deviation of 400 ppm. The signal drift rate was 400 ppm per month.

**Figure 11:**
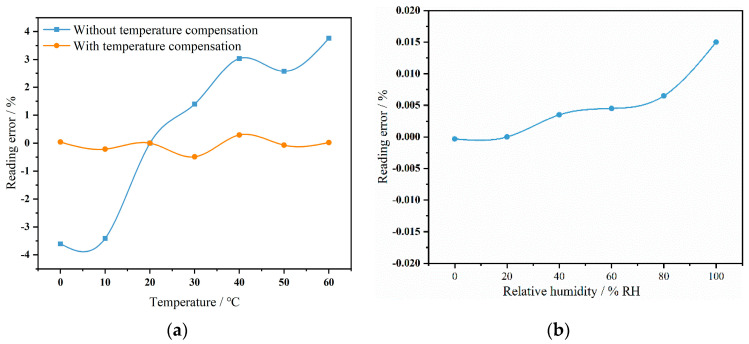
(**a**) Temperature effect on the sensor curve. (**b**) The effect of humidity on the sensor.

The sensor was placed in a constant temperature and humidity chamber to simulate the effects of environmental temperature and humidity changes on the sensor by adjusting different temperatures and humidity levels. The temperature in the chamber was programmed to vary from 0 °C to 60 °C in 10 °C increments. Once the temperature reached the set point and stabilized for half an hour, the sensor signal at the same concentration was collected. The output signal at 20 °C was chosen as the reference point to analyze the signal deviation at different temperatures. The results are shown as the black curve in Figure 11a. At a low temperature of 0 °C, the sensor error was −3.5%, whereas at a high temperature of 60 °C, the error was 4%. This indicates that the sensor’s measurement error reached several thousand ppm, significantly impacting the accuracy of the sensor’s measurements. Therefore, temperature compensation is necessary to bring the sensor’s measurement error back within the normal range. The same temperature compensation algorithm as described in Chapter 3 was applied, establishing a temperature–output relationship model. Signals at different concentrations were tested within the 0–60 °C range, using the signal at 20 °C as the lookup reference. The obtained output signals were used to establish the following function model:

(6) $$∆V\left(T\right)=1.705\times 10^{-7}T^{4}-2.16\times 10^{-5}T^{3}+0.00082T^{3}-0.005T+0.09256$$

(7) $$V=V_{T}+\Delta V\left(T\right)$$

where Δ*V* represents the voltage deviation caused by a certain temperature *T*. The model was used to calculate the deviation in the sensor signal caused by temperature changes. The calibrated voltage value *V* for a specific concentration based on the actual voltage value *V_T_* at the current temperature was calculated, thus mitigating the impact of temperature on the measurements. The relative error curve after temperature compensation is shown as the red curve in Figure 11a. By comparing the red and black curves, it is evident that the errors caused by temperature changes were effectively controlled. The current sensor error, after analysis, was ±27 ppm, which is consistent with the measurement error at a normal temperature. After applying temperature compensation, the sensor maintained the same measurement accuracy within the 0–60 °C range.

The effect of humidity on sensor performance was then evaluated. Keeping the temperature constant, the humidity inside the constant temperature and humidity chamber was adjusted from 0%RH to 100%RH in increments of 20%RH. The fluctuation of the sensor’s output signal was observed to analyze the impact of humidity on sensor performance. As shown in Figure 11b, using the value at 20%RH as a reference, the maximum relative error of the sensor during the entire humidity test was 0.017%, occurring at 100%RH, with a fluctuation of 27 ppm. The sensor’s measurement fluctuations remained within the normal measurement error range. This stability is attributed to the waterproof and breathable membrane covering the sensor’s air vent, which reduces the likelihood of water vapor entering the measurement chamber. Additionally, at around 4.26 μm, the absorption of water vapor is three orders of magnitude weaker than that of CO_2_ gas. Therefore, humidity changes have a limited impact on the sensor’s measurements, preventing significant signal fluctuations.

In coal mines and underground detection, gases such as CH_3_ and CO are often present. Therefore, tests were conducted on the interference gases for the sensor, and the results are shown in Table 2. The tests revealed that although gases such as CH_3_ and CO are present, the sensor exhibited good selectivity. Moreover, these gases do not absorb infrared radiation at 4.26 μm, indicating that the stability of the sensor’s gas concentration detection is unaffected by changes in thermal conductivity. This characteristic ensures that the sensor remains reliable in complex environments.

Table 3 presents a comparison between the current work and various other NDIR systems. The main advantages of this study include high sensitivity, simple fabrication methods, high detection resolution, a small size, and a fast response time.

## 4. Conclusions

This paper presents the development of a high-sensitivity NDIR CO_2_ gas sensor designed for high-precision portable gas analyzers. The overall structure of the infrared CO_2_ gas sensor has been optimized, and a mirror optical system with dual ellipsoidal mirrors has been proposed. Based on this design, an optical gas chamber with a coupling efficiency of up to 54% and a low-noise hardware circuit were developed, and the performance of the miniature infrared CO_2_ gas sensor was validated. The sensor dimensions were 20 mm × 10 mm × 4 mm (length × width × height), which is only 15% of the size of traditional NDIR gas sensors with equivalent detection resolution. The test results show a detection range of 0 to 10%, a sensitivity of 15 μV/ppm, a resolution of 30 ppm, and a detection limit of 90 ppm. The response time was 3 s, and the minimum power consumption was 6.5 mW. This sensor is suitable for high-precision portable analyzers. To achieve better sensor performance and meet the needs of higher-precision detection, the method of adding a reference detector with a 4.15 µm filter can be adopted in the future to monitor emitter power and temperature changes, thereby improving the detection accuracy of the sensor.

## Figures and Tables

**Table 1:** Simulation parameters of the micro-optical gas cell.

Parameter Name	Parameter Value
Initial optical power of the emitter	1 W
Radiation area of the emitter	1.0 × 1.0 mm^2^
Detection area of the detector	1.2 × 1.2 mm^2^
Number of rays	100,000,000
Reflective surface material properties	gold
Number of reflections	≤5

**Table 2:** Selectivity of the NDIR CO_2_ gas sensor towards different gases.

Gas (100 ppm)	Response/mV
CO_2_	32
CO	0.1
NH_3_	0.07
CH_4_	0.05

**Table 3:** Comparison among different NDIR CO_2_ gas sensors.

Authors	Sensitivity/μV/ppm	Range/ppm	Resolution/ppm	*t*_90_/s	LOD/ppm	Size
Vincent [8]	4	0~48,000	250	1.3	/	Large
Jane [14]	4.5	0~200,000	/	23		Large
Shen [26]	/	500~45,000	80	>10	/	Large
Akram [27]	3.3	5000~60,000	25	10	/	Large
Niklas. [28]	7.14	0~200,000	445	/	1200	Large
Jia [11]	0.1	0~100,000	250	2.8	750	Miniature
This work	15	0~100,000	30	3	90	Miniature

## Data Availability

Data are contained within this article.
